# Comparative analysis of proficiencies of various textures and geometric features in breast mass classification using k-nearest neighbor

**DOI:** 10.1186/s42492-021-00100-1

**Published:** 2022-01-12

**Authors:** Harmandeep Singh, Vipul Sharma, Damanpreet Singh

**Affiliations:** 1 Department of Computer Science and Engineering, IKG Punjab Technical University, Jalandhar, Punjab 144603 India; 2 Department of Computer Science and Engineering, Sant Longowal Institute of Engineering and Technology, Sangrur, Punjab 148106 India

**Keywords:** Mammography, Breast cancer, Machine learning, Classification

## Abstract

This paper introduces a comparative analysis of the proficiencies of various textures and geometric features in the diagnosis of breast masses on mammograms. An improved machine learning-based framework was developed for this study. The proposed system was tested using 106 full field digital mammography images from the INbreast dataset, containing a total of 115 breast mass lesions. The proficiencies of individual and various combinations of computed textures and geometric features were investigated by evaluating their contributions towards attaining higher classification accuracies. Four state-of-the-art filter-based feature selection algorithms (Relief-F, Pearson correlation coefficient, neighborhood component analysis, and term variance) were employed to select the top 20 most discriminative features. The Relief-F algorithm outperformed other feature selection algorithms in terms of classification results by reporting 85.2% accuracy, 82.0% sensitivity, and 88.0% specificity. A set of nine most discriminative features were then selected, out of the earlier mentioned 20 features obtained using Relief-F, as a result of further simulations. The classification performances of six state-of-the-art machine learning classifiers, namely k-nearest neighbor (k-NN), support vector machine, decision tree, Naive Bayes, random forest, and ensemble tree, were investigated, and the obtained results revealed that the best classification results (accuracy = 90.4%, sensitivity = 92.0%, specificity = 88.0%) were obtained for the k-NN classifier with the number of neighbors having k = 5 and squared inverse distance weight. The key findings include the identification of the nine most discriminative features, that is, FD26 (Fourier Descriptor), Euler number, solidity, mean, FD14, FD13, periodicity, skewness, and contrast out of a pool of 125 texture and geometric features. The proposed results revealed that the selected nine features can be used for the classification of breast masses in mammograms.

## Introduction

Breast cancer continues to be one of the deadliest diseases. It is caused by the invasion of abnormal cells across the usual boundaries due to uncontrolled growth and division [1]. According to the latest statistics, female breast cancer remains a significant hurdle, with an estimated 2.26 million new cancer cases, accounting for nearly 24.5% of the 9.22 million new cancer cases diagnosed among women in 2020. Breast cancer has surpassed lung cancer in terms of the cause of mortality among women, accounting for 15.5% of the total 4.43 million deaths in women of all age groups due to cancer [2]. Early detection of breast cancer is the only entity that can help reduce the death rate [3]. Screening using mammogram images is still considered the best, most reliable, and economical method for the detection of early signs of breast cancer. Radiologists must carefully examine mammogram images to detect abnormalities [4]. However, the success and widespread adoption of mammography has drastically increased the workload of radiologists. Due to this increased workload, even expert radiologists can miss a considerable number of abnormalities or can misinterpret abnormalities that may increase the number of false-positive and false-negative reports. To resolve these issues, computer-aided diagnosis (CAD) systems are used by radiologists as secondary readers [5]. Generally, radiologists look for four different types of abnormalities in mammogram images, namely masses, microcalcifications, architectural distortions, and asymmetric breast tissues, as early signs of breast cancer [6]. Among these, masses and microcalcifications are the most frequently occurring types of abnormalities, any other types of abnormalities are usually found in rare cases. It should also be noted that the diagnosis of masses is a more challenging task than that of micro-calcifications [3]. Moreover, successful CAD systems have already been clinically approved for the diagnosis of microcalcifications. As a result of this, CAD systems for breast masses are attracting considerable research interest.

Generally, the diagnosis of breast masses involves the detection and classification of breast masses. Masses are generally characterized by their shape, margin, and texture. Benign masses possess round and oval shapes with well-circumscribed and smooth boundaries as opposed to malignant masses, which usually possess irregular shapes with rough, ill-defined, and speculated boundaries [7]. Significant differences can also be seen between the texture of benign and malignant masses, with the former being mostly smooth and homogeneous and the latter having a heterogeneous and rough texture [8].

So far, numerous researchers in the literature have made significant contributions to the analysis of texture and geometry-based features. For instance, Mudigonda et al. [9] compared the effectiveness of two sets of features, gradient-based and texture-based, for the classification of breast masses. The best classification accuracy of 82.1% with 0.85 as an area under the receiver operating characteristics curve has been reported by using gray-level co-occurrence matrix (GLCM)-based texture features with a posterior probability-based classifier for the Mammographic Image Analysis Society (MIAS) database. Yang et al. [10] developed a two-stage CAD system for the detection and classification of breast masses. In the first stage, the statistical gray-level difference matrix and fractal dimension-based five texture features were used for the detection and extraction of breast masses using a probabilistic neural network (PNN). In the second stage, four shape features were further coupled with the previously used five texture features for classification using a PNN and achieved an accuracy of 84.1% for the mammograms taken from Taichung Veteran General Hospital. Kegelmeyer et al. [11] proposed a CAD system for the detection of speculated mass lesions by using four Laws’ texture features with a new feature responsive to stellate patterns. A sensitivity of 97.0% with 0.28 FP per image has been reported. Nandi et al. [12] employed a set of 22 features related to shape, texture, and edge sharpness for the classification of breast mass lesions using genetic programming classification techniques that implicitly possess feature selection capability. A shape-based feature called fractal concavity was the most discriminative feature among all, and the proposed system showed classification accuracies above 99.5% and 98.0% for the training and testing sets, respectively. Delogu et al. [13] developed a CAD system for the segmentation and classification of breast masses in mammograms. To extract the exact mass lesions, the first region of interest (ROI) containing the mass lesions was located by expert radiologists, and then the wavelet transform-based segmentation technique was used to separate the mass lesions from the normal tissues in the ROI. Repeated experiments were performed with various combinations of 16 shape-, size-, and intensity-based features using a multi-layered perceptron neural network classifier. The best classification results were obtained using the 12 most powerful features out of a total of 16 computed features. Domínguez and Nandi [14] conducted various experiments to explore the usefulness of a set of six mass margin characterization features extracted from simplified versions of contours. The performance of each of these features and their various combinations were evaluated using three different classifiers on a set of mammographic images taken from mini-MIAS and Digital Database for Screening Mammography (DDSM) datasets. It was found that out of all the possible sets of features, spiculation features performed the best, and most of the systems formed by using different combinations of features, datasets, and classifiers were more efficient in identifying benign masses than malignant masses. Ganesan et al. [15] presented a classification pipeline for studying the textural changes that occurred in mammogram images of cancerous breasts and further improved the classification accuracy. Features based on higher-order spectra, local binary patterns, Law’s texture energy, and discrete wavelet transform were extracted from the manually segmented mass lesions. Out of the six classifiers used, the decision tree (DT) classifier showed promising results. Sharma and Khanna [16] showed that the Zernike moment of order 20 performed better than the other texture descriptors, spatial grey-level co-occurrence matrices (SGLCM), and discrete cosine transform, with a support vector machine (SVM) classifier. The proposed system attained 99.0% sensitivity and 99.0% specificity with the image retrieval in medical applications dataset and 97.0% sensitivity and 96.0% specificity with the DDSM dataset. Liu and Tang [17] investigated the classification performance of a CAD system by employing several feature selection algorithms with an SVM classifier. A new feature selection algorithm called SVM-based recursive feature elimination with normalized mutual information feature selection has been proposed for the selection of an optimal set of features out of a total of 31 features (12-geometry and 19-texture). Experiments were carried out with 826 ROIs (408 m and 418 b) taken from the DDSM dataset. The best area under curve (AUC) values of 0.9439 and 0.9615 were achieved with the proposed feature selection technique and SVM classifier with ten-fold cross-validation and leave-one-out scheme, respectively. Kashyap et al. [18] proposed a CAD system for the diagnosis of breast masses in mammograms and their shape analysis. The fast fuzzy C-means clustering algorithm was employed for the extraction of mass lesions from pre-processed mammograms. SVM was used to classify segmented ROIs as mass or non-mass using the texture features. The proposed system was evaluated on two datasets, mini-MIAS and DDSM, and achieved the highest sensitivity, specificity, accuracy, and AUC values of 91.7%, 96.2%, 95.4%, 96.2%, and 94.6%, 92.7%, 92.0%, 95.3% respectively. Finally, shape analysis was performed by employing radon transform-based features. Lbachir et al. [19] proposed a complete CAD system for breast masses. A histogram region analysis-based k-means algorithm has been proposed for the segmentation of breast mass lesions from enhanced mammogram images. Texture and shape features were then used for false-positive reduction with the bagged trees classifier. Finally, SVM was employed for the classification of breast masses. The proposed system achieved 93.1% and 90.8% detection accuracies and 94.2% and 90.4% classification accuracies for the MIAS and the curated breast imaging subset of DDSM datasets, respectively. Hosni et al. [20] used a systematic map to examine the state-of-the-art ensemble classification methods when applied to breast cancer in terms of nine factors: publication venues, medical tasks addressed, empirical and research types used, types of ensembles proposed, single techniques used to construct the ensembles, validation framework used to evaluate the proposed ensembles, and the tools used. The goal of this study is to conduct a systematic mapping investigation of single approaches. Al-Antari et al. [21] used a You Only Look Once detector for the detection of breast lesions and deep learning convolutional models for retrieving deep features. Classification accuracies of 94.5%, 95.8%, and 97.5%, respectively, for the DDSM dataset and 88.7%, 92.5%, and 95.3%, respectively, for the INbreast dataset were achieved by employing three modified deep learning classifiers, namely regular feedforward convolutional neural network, ResNet-50, and InceptionResNet-V2.

Most of the aforementioned studies dealt with either mass classification or feature selection techniques. Textures and geometric features are being used by most researchers for the characterization of breast mass lesions so they can be classified into benign and malignant categories. It is a well-established fact that usually, all extracted features do not contribute equally to the classification of masses, and some features perform better in combination with other features. Therefore, it is interesting to identify the significant contributing features from the pool of total extracted features. Feature selection algorithms are generally used for the selection of an optimal and relevant subset of extracted features; however, these algorithms cannot be used for performing a comparative analysis of the discriminative capabilities of individual features. There are very few studies in the literature that show a comparative analysis of discriminative capabilities of an individual or a group of features. As a primary contribution, this work is intended to analyze the discriminative capabilities of various texture and geometric (shape and margin) features in CAD systems by incorporating various combinations of texture and geometric features and pattern classification methods. As the key finding, this research investigation revealed the nine most discriminative features out of a pool of 125 texture and geometric features.

The remainder of this paper proceeds as follows. The methods are presented in Section 2. The results are reported and discussed in Section 3, and Section 4 presents the conclusions.

## Methods

**Fig. 1:**
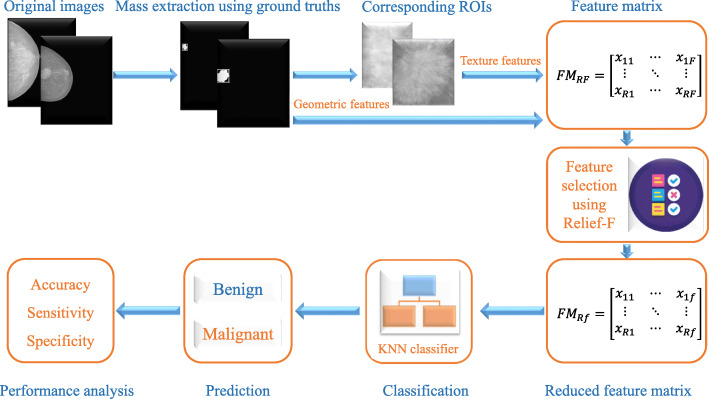
The schematic diagram of the proposed CAD system

In this study, a CAD system was proposed for carrying out a comparative analysis of the proficiencies of various texture and geometric features for the classification of breast masses into benign and malignant categories. The schematic diagram of the proposed CAD system is shown in Fig. 1, which consists of five main stages: arrangement of the mammographic dataset, exact mass lesion extraction, feature extraction, feature selection, and classification. A brief description of each step is provided in the following subsections.

### Mammographic dataset

Fully field digital mammographic (FFDM) images taken from the INbreast dataset were included in this study for carrying out the experiments. All images in the INbreast dataset have a Digital Imaging and Communications in Medicine format and have been acquired at two different resolutions, 3328 × 4084 and 2500 × 3328, using MammoNovation Siemens FFDM equipment at Centro de Mama - Hospital de S. João (CHSJ), breast center Porto. Boundary points in the form of pixel coordinates inscribing various types of abnormalities in mammographic images of breasts are provided with the dataset [22]. A detailed description of the images included in this study for carrying out the experiments is presented in Table 1.

**Table 1:** Detailed description of mammographic image dataset [22]

Total number of images included	Images containing a single mass	Images containing double masses	Images containing three masses	Total number of mass lesions	Number of benign masses	Number of malignant masses
106	98	7	1	115	52	63

**Fig. 2:**
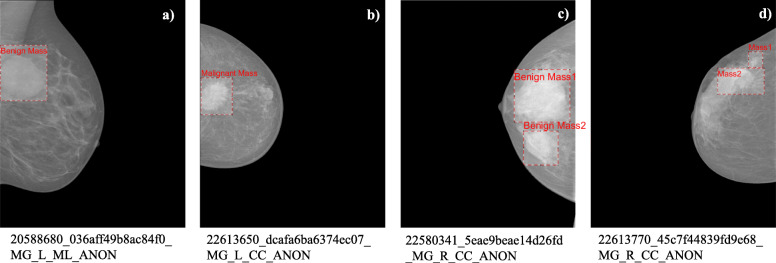
Sample of mammogram images containing benign and malignant masses. (**a**) Single benign mass; (**b**) Single malignant mass; (**c**) Two benign masses; (**d**) Two malignant masses [22]

The samples of mammogram images containing benign and malignant mass lesions are shown for reference in Fig. 2. Figure 2(a) contained a single benign mass lesion, Fig. 2(b) contained a single malignant mass lesion, Fig. 2(c) contained two benign masses, and Fig. 2(d) contained two malignant masses. Each mass lesion and its corresponding ROI region have been highlighted in the sample mammogram images with labels.

### Methodology

#### Exact mass lesion extraction

**Fig. 3:**
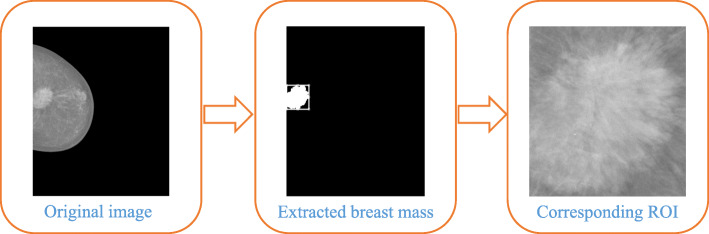
Snapshots for describing the steps used in the extraction of mass lesions

One of the stringent requirements for the extraction of geometric features is the delineation of the exact shapes of breast masses. Delineation of the exact shape of a mass lesion is a complex task and remains a challenging issue. Since the main objective of this study is to investigate the effectiveness of various textures and geometric features in the classification of breast masses, no emphasis is placed on segmentation techniques in this study, and pixel-level ground truth annotations provided with the INbreast dataset have been used for the extraction of the exact shape of the mass lesions. Exact mass lesions were segmented out from the mammogram images by converting the lesion annotations into bounding boxes. Figure 3 illustrates the steps used in the extraction of mass lesions with intermediate results.

#### Feature extraction

After extraction of the exact shapes of breast masses from mammogram images, the next crucial step is the characterization of breast masses. In many CAD systems described in the literature, various combinations of shape, boundary, and texture features have been used for the characterization of breast masses. In this study, textures and geometric (shape and/or margin) features were employed for the characterization of breast masses. The following subsections describe the various textures and geometric features employed in this study for the classification of malignant and benign breast masses.

##### Texture features

Texture analysis is a source of important discriminatory characteristics or features related to the visual patterns of an image. To date, numerous texture feature extraction techniques have been proposed in the literature. These techniques can be broadly grouped into four different approaches: structural, statistical, model-based, and transform methods. Of these, statistical-based feature extraction techniques are the most widely used techniques in the literature. However, it is worth mentioning that these days, it has become difficult to apply feature extraction techniques to any of these approaches because of their increased complexity. Most feature extraction techniques can be categorized into several groups [23]. Table 2 presents the various texture models and the corresponding texture features extracted in this study. The following subsections describe the various texture feature extraction techniques employed in this study.

**Table 2:** Features extracted using different texture models

Model	Extracted features
SGLCM [F1-F14]	‘ASM’, ‘Contrast’, ‘Correlation’, ‘Sum_Squares’, ‘Inverse_Diff_Moment’, ‘Sum_Average’, ‘Sum_Variance’, ‘Sum_Entropy’, ‘Entropy’, ‘Diff_Variance’, ‘Diff_Entropy’, ‘Info_Measure1’, ‘Info_Measure2’, ‘Max_Corr_Coff’
Gray level difference statistics (GLDS) [F15-F19]	‘Homogeneity’, ‘Contrast’, ‘Mean’, ‘Energy’, ‘Entropy’
First order statistical (FOS) [F20-F23]	‘Mean’, ‘Variance’, ‘Skewness’, ‘Kurtosis’
Statistical feature matrix (SFM) [F24-F27]	‘Mean’, ‘Variance’, ‘Skewness’, ‘Kurtosis’
Law’s texture energy measures (LTEM) [F28-F41]	‘EE’, ‘SS’, ‘WW’, ‘RR’, ‘EL’, ‘SL’, ‘WL’, ‘RL’, ‘SE’, ‘WE’, ‘RE’, ‘WS’, ‘RS’, ‘RW’
Fractal [F42-F43]	‘H1’, ‘H2’
Fourier power spectrum (FPS) [F44-F45]	‘Sr’, ‘Stheta’

SGLCM: It has been proven in the literature that second-order statistics perform better than the human visual system in the discrimination of certain classes of textures. Haralick et al. [24] in 1973 proposed a framework for the computation of second-order statistics of aerial images. In second-order statistical texture analysis, texture information is extracted from the grey-level images by calculating the probabilities of occurrence of pairs of gray levels at random distances and four different orientations over an entire image. The extracted texture information is represented in the form of a matrix called GLCM or grey-tone spatial dependence matrix. Since each pixel of an image has eight nearest neighbors except for pixels at the periphery, four GLCMs will be required to represent the second-order statistical texture information in each of the four directions, and these four matrices are denoted as (P_H_ = 0^0^) for the horizontal direction, (P_V_ = 90^0^) for the vertical direction, (P_RD_ = 45^0^) for the right diagonal, and (P_LD_ = 135^0^) for the left diagonal [25, 26]. In this study, 14 SGLCM features presented in Table 2 were extracted and used for the classification of breast masses in mammogram images.

GLDS: GLDS plays an important role in the texture analysis of images by analyzing the local image properties calculated at each point of the given image. In this technique, the image texture is analyzed by considering the class of properties based on absolute differences between pairs of gray levels. To depict the GLDS mathematically, *I*(*x*, *y*) is used to represent the image intensity function, and δ = (Δx, Δy) is used to represent the small displacement [27]. Therefore, for a given small-displacement δ = (Δx, Δy), the difference in gray levels is represented as:

1 $$ {I}_{\updelta}\left(x,y\right)=\mid I\left(x,y\right)-I\left(x+\Delta \mathrm{x},\mathrm{y}+\Delta \mathrm{y}\right)\mid $$

Let p_δ_ denote the probability density of *I*_δ_(*x*, *y*). Therefore, if a given image contains m-different gray levels, then p_δ_ can be represented by an m-dimensional vector whose i^th^ element represents the probability that *I*_δ_(*x*, *y*) will have a value of i. The values of p_δ_ can be easily computed by counting the number of occurrences of each value of *I*_δ_(*x*, *y*) for small integer values of displacements Δx and Δy. The concentration of values in the m-dimensional vector p_δ_ varies according to the variation in the values of *δ* compared to the texture element size. The measures used in this study for the classification of malignant and benign masses include the mean, contrast, entropy, and homogeneity.

FPS: Compared to spatial methods, FPS is preferably used for describing the directionality of periodic texture patterns in images because spatial methods cannot distinguish their global texture patterns because of their local nature. FPS can be used for the extraction of texture primitives by computing the sample power spectrum [28]. The sample power spectrum is denoted by ϕ, and is computed as follows:

2 $$ \phi \left(u,v\right)=F\left(u,v\right){F}^{\ast}\left(u,v\right)={\left|F\left(u,v\right)\right|}^2 $$

Here, F is the Fourier transform of the given image, and F^*^ stands for the complex conjugate of F. FPS features used in this study include the radial sum and angular sum.

FOS features: In the FOS texture analysis, texture measures were computed from the histogram of an image. The histogram of an image is a compendious summary of statistical information stored in an image [23]. The simplicity of this technique lies in the fact that this technique mainly uses the frequency of a particular gray-level intensity for the computation of texture measures and does not consider the correlation between pixels. For any image I having N distinct gray levels and a total of M pixels, the histogram represents the number of pixels in the whole image with gray-level intensity i for each intensity level, and the probability density of occurrence of that intensity level can be calculated as:

3 $$ P(i)=\frac{N(i)}{M} $$

where *N*(*i*) is the total number of pixels with gray-level intensity ‘*i*’. Histogram shapes can be used to draw a number of inferences related to the characteristics of a given image. The parameters used in this study include the mean, variance, skewness, and kurtosis.

SFM: It has been found that most spatial gray-level dependence methods use a single fixed inter-pixel distance for the extraction of second-order statistical texture features. Owing to this constraint, these methods cannot discriminate all the visual texture pairs within an image. Therefore, to discriminate all such visual pixel pairs, a set of inter-pixel distances instead of a single fixed distance has to be used. SFM is one such technique, in which the statistical properties are computed for various inter-pixel distances within an image. In the SFM technique, the matrix can easily be expanded and the size of the matrix does not depend on the number of gray levels but varies according to the inter-pixel distance. In addition, some physical properties can be directly evaluated from the SFM. In this study, SFM was used for the extraction of four texture properties: periodicity, contrast, coarseness, and roughness [29].

**Fig. 4:**
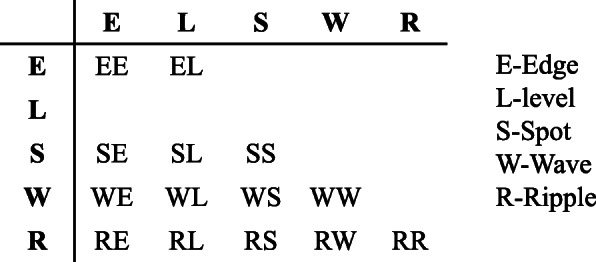
Masks employed in the extraction of law’s texture energy measures [30]

LTEM: LTEM is a technique used to measure the amount of texture variation within a window of fixed size. In this technique, 1-dimensional local convolutional masks were used for the extraction of the texture features. This technique uses three simple vectors of length 3 to drive the law’s texture energy measures [23, 28, 30]. Furthermore, we obtained five vectors of length 5 after the convolution of three vectors with themselves or with each other. Law masks of 5 × 5 dimensions were then obtained by multiplying these vectors with themselves or with each other. One of these vectors should be a column vector of length 5, and the other should be a row vector of the same length. Finally, texture features in the form of statistical values are calculated from the set of energy images obtained from the convolution of masks with the given image. In this study, we used a total of 14 masks, as presented in Fig. 4, for the extraction of law’s texture measures.

Fractal texture analysis: Mandelbrot [31] in 1977 proposed the concept of fractals for describing complex structures that cannot be described using traditional Euclidean geometry. Natural objects generally have complex and irregular structures that cannot be adequately represented by shapes such as spheres, cylinders, and cubes. Fractal analysis is a compendium and precise method for representing complex patterns that recur at various scales and resolutions. Such complex objects usually possess the property of self-similarity, and this is one of the central concepts of fractal geometry. It has been found in the literature that features derived from fractal analysis play an important role in distinguishing between malignant and benign breast masses as malignant breast masses possess more complex textures than benign masses [7, 32]. The two most commonly used techniques for calculating the fractal dimension are the box-counting method and the fractional Brownian motion model [33]. In this study, Hurst coefficients H1 and H2, calculated using the fractional Brownian motion model at two different resolutions, were used as texture features for the classification of breast masses [28].

##### Geometry features

The shape and margin of breast masses in mammograms are important indicators of malignancy, and the same can be used for the diagnosis of breast masses using CAD-based systems. Geometry-based approaches are widely used for extracting shape and margin features. It is a well-established fact that both malignant and benign masses originate from a single spot and take different shapes and margins after growing circumferentially. Generally, benign masses attain round and oval shapes with well-circumscribed and smooth boundaries, while malignant masses attain irregular shapes with rough, ill-defined, and speculated boundaries [34].

One of the stringent requirements of geometry-based approaches is that they require the exact delineation of mass shapes. However, delineating the exact mass from mammogram images is a complex task and has always remained a challenging issue. Despite these problems, shape and margin features still have great significance in the classification of breast masses. The various shape and margin features used in this study are listed in Table 3.

**Table 3:** Various shape and margin features

Features models	Feature index
Shape features	F46-F58 (area, major axis length, minor axis length, eccentricity, orientation, convex area, filled area, Euler number, equiv. diameter, solidity, extent, perimeter, perimeter cirratio)
Zernike moments	F59-F73
Fourier descriptors	F74-F125

In addition to shape features, Zernike moments and Fourier descriptors were also included in this study. Zernike moments can be described as a set of descriptors computed by mapping an image over a set of complex Zernike polynomials [16]. Zernike moments have been widely used in the literature to characterize the shape features of breast masses. The widespread popularity of Zernike moments is due to their orthogonality and the fact that these moments are invariant to an arbitrary rotation of the describing shape. Therefore, these descriptors can be used to describe the shape characteristics, regardless of the rotation of the mass with minimum information redundancy. Zernike moments of order n with repetition m for any continuous image function *f*(*x*, *y*) are computed as follows:

4 $$ {Z}_{nm}=\frac{n+1}{\pi }{\sum}_x{\sum}_yf\left(x,y\right){\left[{V}_{nm}\left(x,y\right)\right]}^{\ast } $$

where *V*_*nm*_(*x*, *y*) is the set of orthogonal polynomials defined on the unit disk ( *x*^2^ + *y*^2^ ≤ 1) using radial polynomials and ‘∗’ denotes the complex conjugate. Higher-order Zernike moments suffer from the problem of high computational complexity and higher sensitivity to noise, but these can act as better shape descriptors compared to low-order Zernike moments with careful selection.

Although Zernike moments are robust in performance, they also suffer from several limitations. The first problem with Zernike moments is that the pixel coordinates must be mapped to the range of the unit circle. The second problem with Zernike moments is that the radial and circular features computed by Zernike moments lie in different domains and are not consistent with each other. The third problem with Zernike moments is related to the calculation of the circular spectral features. These features are not calculated evenly for each order because important shape descriptors can be lost.

To overcome these limitations, generic fourier descriptors (GFD), proposed by Zhang and Lu [35], were included in this study. The GFDs are extracted by applying a 2-D Fourier transform to a polar-raster sampled shape.

5 $$ {PF}_2\left(\rho, \phi \right)={\sum}_r{\sum}_if\left(r,{\theta}_i\right)\mathit{\exp}\left[j2\pi \left(\frac{r}{R}\rho +\frac{2\pi i}{T}\phi \right)\right] $$

where 0 ≤ *r* ≤ *R* and *θ*_*i*_ = *i*(2*π*/*T*)(0 ≤ *i* < *T*); 0 ≤ *ρ* < *R*, 0 ≤ *ϕ* < *T*. *R* is the radial frequency resolution, and *T* is the angular frequency resolution. The normalized coefficients are the GFDs. The city block distance between two GFDs corresponding to different shapes was used to determine the similarity between the two shapes [36]. GFDs offer several advantages over Zernike moments. The first advantage is that the computed features are pure spectral features and are simpler to compute. Second, owing to their capability of multi-resolution analysis in both radial and circular directions of the shape, GFDs exhibit better retrieval performance.

#### Feature selection

A total of 125 texture and geometric features were computed in this study. However, many real-time pattern recognition applications with large feature sets may suffer from performance degradation owing to the presence of redundant and irrelevant features. Feature selection is a data pre-processing step employed for the selection of an optimal and relevant set of features. There are mainly two types of feature selection techniques: filter-based techniques and wrapper-based techniques [37]. Filter-based techniques do not require any classifier and they perform feature selection independently; however, wrapper-based feature selection techniques work in conjunction with classifiers and select only those sets of features that optimize the proposed objective function.

The main objective of this study was to determine the most discriminative features from the pool of total features, therefore, a filter-based feature selection technique was employed for the selection of the top 20 most discriminative features out of a pool of 125 features. To select the best feature selection technique, an experiment was carried out by employing four state-of-the-art filter-based feature selection techniques, namely Relief-F [38], Pearson correlation coefficient [39], neighborhood component analysis [40], and term variance [41]. After analysis of the obtained results, the Relief-F feature selection technique was found to be the best feature selection technique for selecting the top 20 most discriminative features in this study.

##### Relief-F

The original relief algorithm proposed by Kira and Rendel [42] was inspired by instance-based learning. Relief assigns each feature a feature score, which can subsequently be used to rank and select the highest-scoring features for feature selection. These scores can also be used as feature weights to aid downstream modeling. The discovery of feature value discrepancies between nearest-neighbor instance pairs was used to score relief features. The feature score is lowered if a feature value difference is discovered in a surrounding instance pair within the same class. The feature score increases if a feature value difference is found in a surrounding instance pair with different class values. The original Relief algorithm is rarely used these days and has been replaced by various successors. Relief-F is the best and the most widely used variant of the relief algorithms. The Relief-F algorithm was proposed by Kononenko [43] and ‘F’ in the Relief-F algorithm suggests that it is the sixth variant [A to F] of the Relief algorithm.

There are four key differences between Relief-F and Relief. Firstly, Relief-F uses a user option k that defines the use of k nearest hits and k nearest misses in the score update for each target instance (rather than a single hit or miss). This refinement improved the accuracy of the weight estimates, especially for noisy problems. Relief-A was the name given to this method modification when it was first proposed. Second, three different solutions for dealing with partial data were offered (i.e., missing data values) under the variant names Relief (B-D). Third, two different solutions for dealing with multi-class endpoints were offered under the variant names Relief-E and Relief-F. Finally, it is assumed that the quality of the weight estimates would improve when the parameter m approaches the total number of occurrences *n*. To add clarity, every instance in the dataset has the opportunity to be the target instance once. The details of the Relief-F feature selection algorithm can be found in publications [44–46].

#### Classification

In this study, the k-NN classifier was employed for the classification of breast mass lesions into malignant and benign categories. The work of k-NN is based on the idea that cases in a dataset are often found in close proximity to others with similar characteristics. If each instance has a classification label, the value of an unclassified instance’s label can be determined by looking at the class of its closest neighbors. The k-NN finds the k closest instances to the query instance and classifies it by finding the most common class label. In general, instances can be considered as points in an n-dimensional instance space, with each n-dimension corresponding to one of the n-features needed to characterize an instance. The relative distance between instances is more important than the absolute position of the instances within this region. A distance metric was used to calculate relative distance. In theory, the distance metric should reduce the distance between two similarly categorized instances while increasing the distance between instances of different classes [47].

Therefore, the performance of k-NN depends on the selection of only two parameters: the number of k nearest neighbors and the distance metric. The value of k should be chosen in such a way that it can result in the highest classification accuracy. Furthermore, distance metrics were used to calculate the nearest distances. Various distance metrics have been proposed in the literature, and the selection of an appropriate distance metric depends on the type and nature of the dataset [48].

## Results and discussion

A comparative analysis was carried out by comparing the performance measures computed for all individual feature models and the stated combinations. This was done to determine which individual features and their various combinations provide better classification performance. The k-NN classifier was used for the evaluation of classification performance by incorporating ten-fold cross-validation, and the performance was observed in the form of mean accuracy, sensitivity, and specificity obtained after ten repetitions. All experiments were performed using MATLAB R2018a software running on a Windows 10 operating system with an Intel(R) Core (TM) i5-8250U CPU@ 1.60 GHz 1.80 GHz with 8 Gb of RAM.

Initially, all 125 features were employed to compute the classification performance of six different state-of-the-art classifiers, namely k-NN [49], SVM [50], DT [51], Naive Bayes (NB) [52], random forest (RF) [53], and ensemble tree (ET) [54] with a 10-fold cross-validation strategy. The experiment was repeated ten times, and the final results were obtained by averaging the results of the ten experiments. The results are presented in Table 4.

**Table 4:** Classification results were obtained for six different state-of-the-art classifiers using a set of all 125 features

Classifier	Accuracy (%)	Sensitivity (%)	Specificity (%)
k-NN [49]	76.0	73.0	80.0
SVM [50]	**80.0**	**78.5**	**82.0**
DT [51]	68.7	70.8	66.0
NB [52]	72.2	64.6	82.0
RF [53]	73.1	80.0	64.0
ET [54]	72.2	73.8	70.0

A detailed analysis of the results is presented in Table 4, it was found that the best classification results (accuracy = 80.0%, sensitivity = 78.5%, and specificity = 82.0%) were obtained with the SVM classifier while using all 125 features. We would like to mention here that although SVM outperformed all other classifiers in the initial experiment, during the subsequent experimentation, it was found that the k-NN classifier had a better performance than the SVM and all other classifiers for various combinations of textures and geometric features, including a reduced set of nine most discriminating features proposed in this study. Based on these results, only the k-NN classifier is presented in the subsequent sections of this study.

Furthermore, in order to investigate the discriminating capabilities of the individual as well as various combinations of textures and geometric features, numerous experiments were carried out in this study. In the first experiment, features extracted by seven different texture models were employed individually to assess their discriminative capabilities in the classification of breast masses by using five different variants of the k-NN classifier. Five variants of k-NN include fine k-NN, medium k-NN, cosine k-NN, cubic k-NN, and weighted k-NN. All these variants differ in two parameters, namely the distance metric and distance weight. The second experiment was carried out using geometric features, Zernike moments, and Fourier descriptors, individually. In the third experiment, three different sets of features, including all textures, all geometric features, and a combination of textures and geometric features, were used for classification by employing five different variants of k-NN. The classification results obtained by employing the seven individual texture models are listed in Table 5.

**Table 5:** Classification results for various texture models

Features	Fine k-NN			Medium k-NN			Cosine k-NN			Cubic k-NN			Weighted k-NN		
	Accuracy (%)	Sensitivity (%)	Specificity (%)	Accuracy (%)	Sensitivity (%)	Specificity (%)	Accuracy (%)	Sensitivity (%)	Specificity (%)	Accuracy (%)	Sensitivity (%)	Specificity (%)	Accuracy (%)	Sensitivity (%)	Specificity (%)
SGLCM	54.0	58.0	49.0	59.0	53.0	68.0	56.0	60.0	51.0	56.0	51.0	64.0	57.0	62.0	50.0
GLDS	53.0	60.0	44.0	47.0	46.0	48.0	61.0	59.0	63.0	48.0	48.0	48.0	54.0	67.0	36.0
FOS	61.0	61.0	62.0	63.0	58.0	68.0	62.0	68.0	55.0	63.0	62.0	65.0	63.0	67.0	57.0
SFM	75.0	82.0	66.0	67.0	69.0	65.0	67.0	68.0	67.0	70.0	72.0	66.0	75.0	83.0	64.0
LTEM	56.0	60.0	51.0	61.0	69.0	51.0	63.0	68.0	59.0	60.0	67.0	52.0	64.0	77.0	48.0
Fractal	75.0	82.0	66.0	60.0	76.0	41.0	55.0	65.0	43.0	61.0	77.0	42.0	71.0	86.0	53.0
FPS	54.0	55.0	52.0	53.0	59.0	46.0	56.0	60.0	51.0	54.0	59.0	48.0	54.0	64.0	42.0

Inspection of the results presented in Table 5 showed that the SFM and fractal features outperform other feature models in terms of all performance metrics. Furthermore, to assess the discriminative capabilities of the geometric features, the classification results using individual geometric feature models are presented in Table 6.

**Table 6:** Classification results for various geometry feature models

Features	Fine k-NN			Medium k-NN			Cosine k-NN			Cubic k-NN			Weighted k-NN		
	Accuracy (%)	Sensitivity (%)	Specificity (%)	Accuracy (%)	Sensitivity (%)	Specificity (%)	Accuracy (%)	Sensitivity (%)	Specificity (%)	Accuracy (%)	Sensitivity (%)	Specificity (%)	Accuracy (%)	Sensitivity (%)	Specificity (%)
SHAPE	57.0	57.0	58.0	54.0	56.0	52.0	54.0	56.0	50.0	54.0	57.0	51.0	57.0	59.0	55.0
ZM	59.0	62.0	56.0	54.0	51.0	56.0	53.0	51.0	56.0	54.0	50.0	58.0	58.0	62.0	53.0
FD	74.0	72.0	77.0	72.0	62.0	85.0	71.0	62.0	85.0	71.0	60.0	85.0	72.0	66.0	80.0

After a detailed analysis of the results presented in Table 6, it was found that Fourier descriptors perform very well in the classification of breast masses in terms of all performance measures (accuracy, sensitivity, and specificity). Shape and Zernike moments also contribute considerably to sensitivity, along with the Fourier descriptors.

Finally, three sets of features consisting of all textures, all geometric features, and a combined set of all textures and geometric features, respectively, were employed for the classification of breast masses using various versions of k-NN. Table 7 presents the classification results in terms of accuracy, sensitivity, and specificity.

**Table 7:** Classification results for three sets of features including all textures, all geometric features, and a combination of texture and geometry

Features	Fine k-NN			Medium k-NN			Cosine k-NN			Cubic k-NN			Weighted k-NN		
	Accuracy (%)	Sensitivity (%)	Specificity (%)	Accuracy (%)	Sensitivity (%)	Specificity (%)	Accuracy (%)	Sensitivity (%)	Specificity (%)	Accuracy (%)	Sensitivity (%)	Specificity (%)	Accuracy (%)	Sensitivity (%)	Specificity (%)
Texture (T)	72.0	78.0	64.0	64.0	63.0	66.0	67.0	74.0	58.0	63.0	68.0	58.0	72.0	77.0	66.0
Geometry (G)	72.0	72.0	72.0	72.0	60.0	86.0	75.0	70.0	82.0	72.0	61.0	86.0	73.0	67.0	81.0
Combined (T&G)	73.0	67.0	82.0	71.0	59.0	88.0	76.0	73.0	80.0	72.0	59.0	89.0	75.0	67.0	84.0

The analysis of the above results showed that the geometric features perform better than the features related to texture, and the overall accuracy improves when using a combination of texture and geometric features. Textures also play a significant role in the identification of positive cases, and hence, contribute considerably to improving the classification performance.

As a primary contribution, this work is intended to identify the most discriminative features from the pool of total features. Four state-of-the-art filter-based feature selection techniques, namely Relief-F, pearson correlation coefficient, neighborhood component analysis, and term variance, were employed to rank the features according to their discriminative capabilities. The top 20 most discriminative features were selected for each of the four feature selection techniques. Table 8 presents the rank-wise lists of the top 20 most discriminative features for each of the four feature selection techniques employed in this experiment.

**Table 8:** Rank-wise lists of the top 20 most discriminative features selected by four different feature selection techniques

Rank	Relief-F		Pearson correlation coefficient		Neighbourhood component analysis		Term variance	
	Index	Feature name	Index	Feature name	Index	Feature name	Index	Feature name
1	F99	FD26	F86	FD13	F1	ASM	F45	Stheta
2	F53	Euler number	F99	FD26	F2	Contrast	F44	Sr
3	F55	Solidity	F87	FD14	F3	Correlation	F51	Convex area
4	F56	Extent	F82	FD9	F4	Sum squares	F52	Filled area
5	F4	Sum squares	F83	FD10	F5	Inverse diff moment	F46	Area
6	F20	Mean	F85	FD12	F6	Sum average	F57	Perimeter
7	F87	FD14	F53	Euler number	F7	Sum variance	F48	Minor axis length
8	F6	Sum average	F92	FD19	F8	Sum entropy	F47	Major axis length
9	F7	Sum variance	F84	FD11	F9	Entropy	F54	Equiv diameter
10	F86	FD13	F95	FD22	F10	Diff variance	F21	Variance
11	F66	ZM3–1	F90	FD17	F11	Diff entropy	F53	Euler number
12	F67	ZM31	F33	SL	F12	Info measure1	F50	Orientation
13	F26	Periodicity	F74	FD1	F13	Info measure2	F7	Sum variance
14	F82	FD9	F97	FD24	F14	Max Corr. Coff	F20	Mean
15	F43	H2	F98	FD25	F15	Homogeneity	F24	Coarseness
16	F21	Variance	F124	FD51	F16	Contrast	F4	Sum squares
17	F22	Skewness	F79	FD6	F17	Mean	F16	Contrast
18	F25	Contrast	F43	H2	F18	Energy	F25	Contrast
19	F8	Sum entropy	F81	FD8	F19	Entropy	F6	Sum average
20	F9	Entropy	F80	FD7	F20	Mean	F23	Kurtosis

Furthermore, to compare the performance of the feature selection algorithms, the top 20 features selected by each of the four feature selection algorithms were passed to the cosine variant of the k-NN classifier with the number of neighbors k = 5 and squared inverse distance weight individually. The classification results obtained for each feature subset are listed in Table 9.

**Table 9:** Classification results obtained using the top 20 features selected by four different feature selection algorithms

Feature selection	Accuracy (%)	Sensitivity (%)	Specificity (%)
Relief-F [38]	**85.2**	**88.0**	**82.0**
Pearson correlation coefficient [39]	80.9	80.0	82.0
Neighbourhood component analysis [40]	75.7	75.0	76.0
Term variance [41]	60.0	66.0	52.0

After an exhaustive analysis of the obtained results, it was observed that the best classification results (accuracy = 85.2%, sensitivity = 88.0%, and specificity = 82.0%) were obtained by using the top 20 features selected by the Relief-F feature selection algorithm. Further investigation of the top 20 most discriminative features selected by the Relief-F feature selection algorithm includes both textures and geometric features. The selected texture features belong to various texture feature models, including the SGLCM model, FOS, SFM, and fractal texture analysis model. The maximum number of features (five) belong to the SGLCM feature model, and the feature names are sum squares, sum average, sum variance, sum entropy, and entropy. The features belonging to the FOS model are mean, variance, and skewness. The SFM features include periodicity and contrast, and the only feature belonging to the fractal texture analysis model is H2. The selected geometric features include features belonging to all three geometric feature models, including shape, Zernike moments, and Fourier descriptors. Shape features include the Euler number, solidity, and extent. The features belonging to the Zernike moments are ZM3–1 and ZM31. The maximum number (four) of the selected geometric features belongs to the Fourier descriptor model, and the feature names are FD26, FD14, FD13, and FD9.

For further investigation, feature selection was applied to a set of textures and geometric features individually, by employing the Relief-F algorithm. The sets of the top 20 most discriminative texture and geometric features selected by employing the Relief-F feature selection algorithm are presented in Table 10.

**Table 10:** Rank-wise lists of the top 20 most discriminative textures and the top 20 most discriminative geometric features selected by the Relief-F feature selection algorithm

Rank	Rank wise top 20 texture features			Rank wise top 20 geometry features		
	Index	Feature name	Feature model	Index	Feature name	Feature model
1	F42	H1	FRACTAL	F99	FD26	Fourier descriptor
2	F43	H2	FRACTAL	F103	FD30	Fourier descriptor
3	F33	SL	LTEM	F82	FD9	Fourier descriptor
4	F27	Roughness	SFM	F84	FD11	Fourier descriptor
5	F7	Sum variance	SGLCM	F66	ZM3–1	Zernike moments
6	F26	Periodicity	SFM	F67	ZM31	Zernike moments
7	F25	Contrast	SFM	F53	Euler number	Shape descriptor
8	F4	Sum squares	SGLCM	F87	FD14	Fourier descriptor
9	F20	Mean	FOS	F86	FD13	Fourier descriptor
10	F6	Sum average	SGLCM	F97	FD24	Fourier descriptor
11	F24	Coarseness	SFM	F70	ZM4–2	Zernike moments
12	F32	EL	LTEM	F72	ZM42	Zernike moments
13	F16	Contrast	GLDS	F74	FD1	Fourier descriptor
14	F15	Homogeneity	GLDS	F92	FD19	Fourier descriptor
15	F19	Entropy	GLDS	F55	Solidity	Shape descriptor
16	F17	Mean	GLDS	F56	Extent	Shape descriptor
17	F37	WE	LTEM	F100	FD27	Fourier descriptor
18	F22	Skewness	FOS	F119	FD46	Fourier descriptor
19	F23	Kurtosis	FOS	F115	FD42	Fourier descriptor
20	F9	Entropy	SGLCM	F104	FD31	Fourier descriptor

Further experimentation was conducted to investigate the classification results obtained by employing the top 20 geometric and texture features individually using six state-of-the-art classifiers. The results are presented in Table 11.

**Table 11:** Classification results obtained by employing the top 20 texture, top 20 geometric, and top 20 combined texture and geometric features with six different state-of-the-art classifiers

Classifier	Feature set	Accuracy (%)	Sensitivity (%)	Specificity (%)
k-NN	Texture features (T)	73.9	83.1	62.0
	Geometry features (G)	77.4	76.0	78.0
	Combined (T&G)	**85.2**	**88.0**	**82.0**
SVM	Texture features (T)	71.3	73.8	68.0
	Geometry features (G)	72.2	73.8	70.0
	Combined (T&G)	**81.7**	**84.6**	**78.0**
DT	Texture features (T)	68.9	69.2	68.0
	Geometry features (G)	71.2	67.6	76.0
	Combined (T&G)	**71.5**	**73.8**	**68.0**
NB	Texture features (T)	67.9	64.6	72.0
	Geometry features (G)	70.4	72.3	68.0
	Combined (T&G)	**77.4**	**75.4**	**80.0**
RF	Texture features (T)	73.9	84.6	60.0
	Geometry features (G)	74.1	75.4	72.0
	Combined (T&G)	**80.6**	**86.1**	**74.0**
ET	Texture features (T)	74.4	83.0	64.0
	Geometry features (G)	76.3	81.5	70.0
	Combined (T&G)	**81.1**	**83.0**	**78.0**

From a detailed analysis of the classification results presented in Table 11, it can be inferred that the best results are obtained when the combinations of textures and geometric features are used as compared to the individual texture and geometric features. Moreover, it can also be observed that the cosine version of the k-NN classifier employed in this experiment with a number of neighbors k = 5 and squared inverse distance weight outperformed all other classifiers using a reduced set of features.

Further experimentation was carried out to investigate all 20 features selected by the Relief-F feature selection method. Table 12 presents the classification performances of the top 20 features when used individually for classification with the cosine variant of the k-NN classifier with the number of neighbors k = 5 and squared inverse distance weight under the same conditions.

**Table 12:** Classification performances of the top 20 features selected by Relief-F when employed individually

Feature name	Accuracy (%)	Sensitivity (%)	Specificity (%)
FD26	67.0	71.0	62.0
Euler number	58.0	62.0	54.0
Solidity	56.0	62.0	48.0
Extent	56.0	62.0	48.0
Sum squares	50.0	58.0	38.0
Mean	47.0	52.0	38.0
FD14	62.0	62.0	62.0
Sum average	52.0	58.0	44.0
Sum variance	60.0	66.0	52.0
FD13	62.0	69.0	52.0
ZM3–1	44.0	52.0	34.0
ZM31	44.0	52.0	34.0
Periodicity	50.0	57.0	42.0
FD9	61.0	66.0	56.0
H2	65.0	69.0	60.0
Variance (FOS)	47.0	54.0	38.0
Skewness (FOS)	51.0	52.0	50.0
Contrast (SFM)	43.0	51.0	34.0
Sum entropy	58.0	66.0	48.0
Entropy	61.0	69.0	50.0

**Fig. 5:**
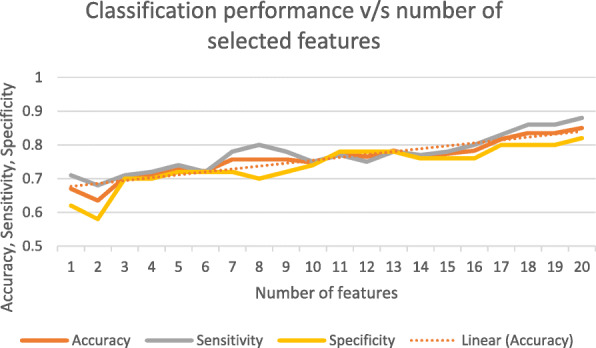
Classification performance (accuracy, sensitivity, and specificity) of Relief-F method versus the number of selected features

From Table 12, it can be easily observed that the FD26 feature extracted using the Fourier descriptor model is the most discriminative feature with a classification accuracy of 67.0%. In addition, it can be observed that as we move downwards, the classification accuracy for individual features continues to decrease, with a few exceptions. Since CAD systems with 67.0% accuracy cannot be used for practical applications, there is a need to include more features to achieve better classification results. Figure 5 presents the curves obtained when the classification performance of different feature subsets of the top 20 features selected by the Relief-F algorithm are plotted against the number of features.

The analysis in Fig. 5 shows that the classification accuracy continues to increase with the addition of a greater number of features with a few exceptions. A similar trend was observed for the sensitivity and specificity. The presence of exceptions demonstrates that some of the features may or may not perform well when used in combination with other features, and they may degrade the classifier’s performance. These exceptions can be eliminated by selecting a highly relevant and optimal set of features.

Therefore, further experiments were performed to solve this problem by investigating the classification performances of manually selected subsets of the top 20 features selected by the Relief-F method. Using the experimental results, a set of nine most discriminating features, that include, FD26, Euler number, solidity, mean, FD14, FD13, periodicity, skewness, and contrast, which yielded higher accuracy, were selected out of the top 20 most discriminating features selected by the Relief-F algorithm. Table 13 presents a list of the selected nine features along with their feature models.

**Table 13:** Experimentally selected top nine most discriminative features out of 20 features selected by Relief-F method

Rank	Feature index	Feature name	Feature model
1	F99	FD26	Fourier descriptor
2	F53	Euler number	Shape descriptor
3	F55	Solidity	Shape descriptor
6	F20	Mean	FOS
7	F87	FD14	Fourier descriptor
10	F86	FD13	Fourier descriptor
13	F26	Periodicity	SFM
17	F22	Skewness (FOS)	FOS
18	F25	Contrast (SFM)	SFM

The best classification results (accuracy = 90.4%, sensitivity = 92.0%, and specificity = 88.0%) were obtained when the proposed set of the top nine most discriminative features was used for classification by employing the cosine variant of the k-NN classifier with the number of neighbors k = 5 and squared inverse distance weight. For validation purposes, the selected set of the nine most discriminative features was used for classification by employing six different state-of-the-art classifiers. The classification results are presented in Table 14.

**Table 14:** Classification results obtained for six different state-of-the-art classifiers using a set of nine most discriminative features

Classifier	Accuracy (%)	Sensitivity (%)	Specificity (%)
k-NN [49]	**90.4**	**92.0**	**88.0**
SVM [50]	86.1	87.7	84.0
DT [51]	73.0	80.0	64.0
NB [52]	74.8	70.8	80.0
RF [53]	78.4	78.4	78.0
ET [54]	81.7	84.6	78.0

A detailed analysis of the results presented in Table 15 showed that the k-NN classifier performed better than other state-of-the-art classifiers in terms of classification accuracy, sensitivity, and specificity. These results support our assertion of proposing the nine most discriminative features out of the pool of 125 features using the k-NN classifier. Finally, the following observations were drawn from the analysis of all the experimental results presented in the above sections:

- Geometric features perform better than textures in the classification of breast masses in mammograms.
- The Fourier descriptor feature (FD26) is listed at the top position among the most discriminative features.
- Out of the seven texture feature models employed in this study, the SFM and FOS feature models perform better than the other texture feature models.
- The cosine variant of the k-NN performs better than the other variants of k-NN.
- No single feature or group of features of the same category can become effective for use in mass classification applications. Therefore, features from different classes must be grouped to achieve the best classification results.
- Although geometric features are more discriminative than texture features, both geometric and texture features need to be combined to obtain the best classification results. In this work, the classification accuracies of the geometric and texture features improved from 75.0% and 67.0%, respectively, to 76.0% when combined.
- Feature selection contributed significantly to improving classification performance. Among the four filter-based feature selection algorithms employed in this study, Relief-F feature selection techniques performed better than the other three algorithms. Classification accuracy further improved from 76.0% to 85.2% by using the top 20 most discriminative features selected by the Relief-F method.
- The classification accuracy showed a rise from 85.2% to 90.4% when using a set of nine experimentally selected features out of the 20 features selected by the Relief-F method.

**Table 15:** Comparison with previous work

Reference	Dataset used	Feature selection	Classifier used	Accuracy (%)
Hans et al. [55]	INbreast	Opposition-based Harries Hawk Optimization	k-NN	78.8
Present study	INbreast	Relief-F	k-NN	90.4

### Comparison with previous works

The present study employs conventional machine learning algorithms, hence, it would be appropriate to compare the results with those of existing studies that have used conventional machine learning algorithms on the INbreast dataset. In Table 15, the results of the present study were compared with a recently published study by Hans et al. [55], in which the authors used conventional machine learning algorithms for feature selection and breast mass classification using the INbreast dataset. In ref. [55], the authors used the Opposition-based Harries Hawk Optimization algorithm for the selection of a reduced set of features out of a set of 54 texture and shape features and the k-NN classifier for the classification of breast masses into malignant and benign categories.

The above results clearly show that the proposed system achieved much higher accuracy than the accuracy achieved in the previous work for the same dataset and by employing the same classifier, that is, k-NN. This improvement in accuracy can be linked to two concepts: in the present study, a wide range of texture and geometric feature models was used for feature extraction and the selection of the nine most discriminating features by employing the Relief-F feature selection algorithm.

## Conclusions

In this study, a comparative analysis of the proficiencies of individual and various combinations of texture and geometric features in the classification of breast masses was conducted. An improved machine learning framework has been proposed, which utilizes a reduced set of the most discriminating textures and geometric features for the classification of breast masses. Total 125 texture and geometric measures were extracted for each of the 115 mass lesions extracted from 106 FFDM images taken from the INbreast dataset. The discriminative capabilities of the individual and various combinations of texture and geometric features were investigated by evaluating the corresponding classification accuracies. To reduce the dimensionality of the extracted feature set, four different state-of-the-art feature selection algorithms, that is, Relief-F, pearson correlation coefficient, neighborhood component analysis, and term variance were applied for the selection of the top 20 most discriminating features. Further experimentation revealed a set of nine most discriminating features out of the 20 features selected by the Relief-F feature selection algorithm, which yielded higher classification accuracy, these included FD26 (Fourier descriptor), Euler number, solidity, mean, FD14, FD13, periodicity, skewness, and contrast. Performance comparisons were conducted among several machine learning algorithms, including k-NN, SVM, DT, NB, RF, and ET. The k-NN with the number of neighbors k = 5 and squared inverse distance weight, outperformed all other algorithms, giving an accuracy of 90.4%, sensitivity of 92.0%, and specificity of 88.0% for the classification of benign and malignant breast masses. The proposed set of features resulted in an improvement in the sensitivity value from 73.0% (without feature selection) to 92.0% (with feature selection), which is the most desired attribute in medical diagnostic systems.

The main objective of this study was to investigate the discriminative capabilities of the individual and different sets of geometric and texture features; therefore, filter-based feature selection techniques have been employed for the selection of the most discriminative top 20 features. The classification results can be further improved using wrapper-based feature selection methods. As a future direction, investigation and use of the latest wrapper-based feature selection algorithms are advised to further improve the classification performance. Further deep learning-based approaches can also be explored for the classification of breast masses in the near future.

## Data Availability

INbreast dataset have been used in this study for experimentation and evaluation of the proposed system and is available in the repository, http://medicalresearch.inescporto.pt/breastresearch/index.php/Get_INbreast_Database

## Abbreviations

ROI: Region of interests
k-NN: k-nearest neighbour
CAD: Computer-aided diagnosis
ROC: Receiver operating characteristics
GLCM: Gray-level co-occurrence matrix
MIAS: Mammographic image analysis society
SGLDM: Statistical gray-level difference matrix
PNN: Probabilistic neural network
DDSM: Digital database for screening mammography
SVM: Support vector machine
AUC: Area under curve
FFDM: Fully field digital mammographic
GLDS: Gray level difference statistics
FPS: Fourier power spectrum
FOS: First order statistical
SFM: Statistical feature matrix
GFD: Generic Fourier descriptors
SGLCM: Spatial grey-level co-occurrence matrices
